# Has the “Double Reduction” policy relieved stress? A follow-up study on Chinese adolescents

**DOI:** 10.1186/s13034-022-00530-6

**Published:** 2022-11-28

**Authors:** Dongfang Wang, Xiao-Yan Chen, Zijuan Ma, Xianchen Liu, Fang Fan

**Affiliations:** 1 Centre for Studies of Psychological Applications, Guangdong Key Laboratory of Mental Health and Cognitive Science, Ministry of Education Key Laboratory of Brain Cognition and Educational Science, School of Psychology, South China Normal University, Shipai Road, Guangzhou, 510631 China; 2 Center for Public Health Initiatives, University of Pennsylvania, Philadelphia, PA USA

**Keywords:** Depression, Anxiety, Adolescents, “Double Reduction” policy, Stress

## Abstract

**Purpose:**

“Double Reduction” Policy requires schools to reduce the burden of excessive homework and off-campus training for Chinese students to reduce their academic stress and promote mental health. We conducted a study in compulsory education students before and after the “Double Reduction” Policy to explore changes in mental health problems and relevant influential factors.

**Methods:**

A total of 28,398 elementary and junior high school students completed both waves of the survey through electronic questionnaires. Depressive symptoms were assessed using the Patient Heath Questionnaire (PHQ-9), and anxiety symptoms were assessed using the Generalized Anxiety Disorder Scale (GDA-7). Demographic information was evaluated at baseline, and “Double Reduction” related factors and negative life events were measured at follow up.

**Results:**

The overall depression and anxiety levels significantly decreased after the “Double Reduction” Policy. Girls, poor parental marital quality, chronic physical illness, and psychiatric family history were related to increased occurrence of mental health. Sleep duration > 8 h/night, reduced homework, more extracurricular activities more time with parents, and reduced academic stress were protective factors against mental health problems.

**Conclusions:**

The “Double Reduction” Policy has improved the mental health symptoms of students to a certain extent. Appropriately increasing sleep time, participating in more extracurricular activities and parental involvement, and reducing the burden of homework are effective ways to promote the development of students’ mental health.

## Introduction

Chinese parents tend to set high expectations for their children because of the higher standards of general education. Consequently, children’s academic pressure increases thus leading to poor health outcomes [1]. The literature has pointed out that academic motivational goals to compete to get good grades and to be rewarded for their performance were positively related to anxiety symptoms [2]. Zhao and colleagues have found that highly stressful educational environments put Chinese children and adolescents at higher risk of elevated anxiety symptoms [3]. Therefore, it is important to pay attention to the of adolescents’ academic stress, which is critical to promote adolescent mental health.

Students often need to complete a lot of homework and attend off-campus training to improve their academic performance. Studies have shown that students who spend more time on homework and after-school tutoring have later bedtime and shorter sleep duration [4, 5]. Meanwhile, they also suffer from more school-related stress and are more likely to give up activities or hobbies to make way for schoolwork [6, 7]. The stress hypothesis [8] echoes the above-mentioned situations. It argued that chronic stressor exposure (e.g., excessive homework and intensive off-campus training) could result in more severe psychopathology among adolescents. A recent study of students in Singapore found more time studying and doing homework was positively related to higher depressive symptoms [9].

The Chinese government attaches great importance to education; all Chinese children and adolescents can have a nine-year free and mandatory education. In recent years, relevant authorities have also been making efforts to reduce academic stress, preventing children and adolescents’ physical and mental health. In July 2021, the Chinese government implemented the “Double Reduction” Policy to reduce the academic burden of these students, relieve parents’ anxiety, and promote an overall healthy educational environment [10]. Specifically, the government requires schools to reduce the burden of excessive homework and off-campus training [10]. This move may relieve students’ academic pressure to a certain extent and improve their mental health. However, no existing studies have examined changes in mental health problems before and after the “Double Reduction” Policy and related predictors.

From April 21st 2021 to May 12th 2021 (Time 1, T1: before the “Double Reduction” Policy was released), we conducted a cross-sectional survey to investigate mental health in elementary and junior high school students in SZ City (a city in southern Guangdong province, China). From December 17 to 26, 2021 (Time 2, T2: after the “Double Reduction” Policy was implemented), we followed these participants again. This follow-up survey was an opportunity to explore how mental health problems changed in response to the “Double Reduction” Policy and to better understand of factors that affected the occurrence of adolescents’ poor mental health. Three specific objectives were: (a) to examine depression and anxiety prevalence rates among elementary and junior high school students at two surveys; (b) to identify the changes in depressive and anxiety symptoms before and after the “Double Reduction” Policy; and (c) to explore the predictors of changing patterns in these two variables.

## Methods

### Study population and data collection

This study was a two-point repeated cross-sectional survey with a nested longitudinal subsample. The first data collection wave was before the “Double Reduction” Policy and the second wave was after the “Double Reduction” Policy. Before this survey, all participants and their caregivers signed the electronic informed consent form. Our team designed a specialized platform for this project to protect data safety. The local education bureau and the department of mental health services of each school assisted in recruiting participants. All students used an anonymized student number that was assigned based on their registration status at school to log in to the platform to complete questionnaires. They were informed that they could feel free to withdraw from the study at any time. This study was approved by the Human Research Ethics Committee of South China Normal University (SCNU-PSY-2021-094). We also open a free psychological distress hotline named “Xinqing” to provide psychological services when participants needed.

The subjects of this study were Chinese students (primary, grades 1–6; junior high school students, grades 7–9) receiving compulsory education from 152 schools in Guangdong province. We did not include grades 1–4 with the concern that they may not well understand the questionnaire due to their young age. The 9th graders were also excluded so as to follow the students for at least 2 waves before they graduated from schools. A total of 89,283 students (grades 5–8) were recruited in the first timepoint (T1), and 77,236 students (grades 5–8) were recruited in the second timepoint. In T2 survey, the students of 5th graders did not participate in T1 survey, because they were still in 4th grade when T1 survey started. Through data integration, a total of 28,542 students participated in all two web-based surveys and provided complete data on all measures. The following exclusion criteria were used to improve data quality: (a) abnormal response time; (b) inconsistent survey contents (e.g., different demographic information); and (c) having current or history of mental health illness that were identified by the caregivers or teachers. We further excluded 144 participants based on the above-mentioned criteria. Consequently, 28,398 participants were included in the analyses. We used *χ*^2^ tests to compare the prevalence of depression and anxiety at T1 between participants who provided available data for both periods and those who had missing data at T2. There was a small but significant difference between these two groups (depression: 12.6% *vs* 10.2%, *χ*^2^ = 106.24, p = 0.034, Cramer’s V = 0.012; anxiety: 9.0% *vs* 7.7%, *χ*^2^ = 42.92, p < 0.001, Cramer’s V = 0.022).

### Measures

#### Mental health indicators

Depressive symptoms were assessed using the Patient Heath Questionnaire (PHQ-9) [11]. It consists of 9 items, responses to which range from 0-not at all, 1-several days, 2-more than half the days, to 3-nearly every day. Higher summed scores indicate higher levels of depressive symptoms. Previous work has suggested 10 as a cut-off to screen clinical depressive symptoms [12]. Psychometric properties of the PHQ-9 have been described in the Chinese population [13]. In this study, PHQ-9 showed good internal consistency in the two surveys, and the Cronbach’s α was 0.90 and 0.92, respectively.

Generalized Anxiety Disorder Scale (GAD-7) was used for screening and diagnosis of anxiety [14, 15]. Seven items were assessed from 0 (not at all) to 3 (nearly every day), with a higher total score indicating greater anxiety symptoms. A cut-off score of 10 was suggested to identify the clinically significant anxiety symptoms [16]. In the present study, Cronbach’s α of the GAD-7 was 0.94 and 0.94 at T1 and T2, respectively.

#### “Double Reduction” policy related measures

Five self-devised questions were used to assess students’ study and living conditions after the “Double Reduction” Policy (i.e., T2) (1) reduced homework, (2) more extracurricular activities, (3) increased physical activity, (4) more time with parents, and (5) reduced academic stress. Each item is scored from 1 (significantly increase/decrease) 3 (no changes). In this study, we recorded the five items into two categories, with the original categories 1 and 2 being combined into a new category (1 = yes, 2 = no). The Cronbach’s α of the five items was 0.82. In addition, sleep duration was assessed with an item (“How much time do you sleep every day during the past 2 weeks?”). This item included five choices: 1 ≤ 5 h, 2 = 5–6 h, 3 = 6–7 h, 4 = 7–8 h, and 5 ≥ 8 h. Sleep duration > 8 h per night was considered as sleep sufficiency in this study [17, 18].

#### Covariates

Demographic information included sex (boys/girls), age, grade (grade 5–8), school types (public school/private school), boarding at school (yes/no), ethnicity (Han [the major ethnic group in China]/others), whether one child or not (yes/no), parental marital status (good/poor [included separated, divorced and widowed]), family income (monthly) (< ¥12,000/¥12,000–¥30,000/ > ¥30,000/unknown), caregivers’ education (below junior high school/senior high school/college or above), chronic physical illness (yes [having at least one of the following: arthritis, angina, asthma, diabetes, visual impairment or hearing problems [19]]/no), and family history of mental disorders (yes/no).

Negative life events over the past 6 months were assessed using the Chinese version of the Adolescent Self-Rating Life Events Checklist (ASLEC) at T2 [20]. The Checklist consists of 27 items covering interpersonal conflicts, academic stress, being punished, personal loss, physical health problems, and others. Participants rated each item on a five-point scale, from 1 (not at all) to 6 (extremely severe). A higher total score indicates the greater severity of stressful life events. The Cronbach’s α of the ASLEC was 0.97 in the current sample.

### Statistical analysis

Analyses were performed using IBM SPSS Statistics for Version 23.0. The McNemar’s test was used to examine differences in the prevalence of depression and anxiety between T1 and T2. The Chi-square test was used to compare the prevalence rates of depression and anxiety between different groups of demographic characteristics. Based on the cut-off scores (i.e., 10) of the PHQ-9 and GAD-7 at T1 and T2, four patterns of symptoms trajectories were established: (1) Persistent: those scores at T1 and T2 were both above the cut-off value, (2) Remission: those scores were above the cut-off value but were below the value at T2 below the cutoff at T2; (3) New-onset: those did not have mental health problems but presented at T2; (4) Resistance: those did not have mental health problems across two periods. This classification has been used in some previous studies [21–23]. Multivariate logistic regressions were used to examine predictors for the occurrence and patterns of depression and anxiety. Our major aim was to explore the risk and protective factors associated with increased likelihood of developing non-resistance. We set the resistance group as the referent group and compared it with the new-onset group. We also explored the influential factors associated with the decreased likelihood of developing remission. Thus, we set the persistent group as the referent group and compared it with the remission group. In the multivariate logistic regression model, odds ratio (OR) and 95% confidence interval (CI) were used to quantify the strength of the association. Considering that our sample size was relatively large, all statistical significance was set to be p < 0.001 (2-sided tests). Adjusted odds ratios in 1.2–1.5 (or 0.7–0.9) and > 1.5 (or < 0.7) were regarded as weak/moderate and strong associations, respectively [24].

## Results

### Sample characteristics

Among 28,398 participants, 14,981 were boys and 13,417 were girls. The mean (SD) age was 12.28 (1.21) years. 13,934 students (49.1%) were from public schools, and 2317 (8.2%) boarded at schools. Other demographic characteristics are shown in Table 1.

**Table 1:** Prevalence of depression and anxiety at two wave surveys by demographics (N = 28,398)

Characteristics	N	Depression (%)		Anxiety (%)	
		T1	T2	T1	T2
Sex					
Boy	14,981	7.1	6.5	5.3	4.7
Girl	13,417	13.0	12.5	9.8	9.8
*χ*^2^		275.44^***^	301.85^***^	212.52^***^	275.70^***^
Grade [age, years]					
Grade 5 [11.14(0.62)]	10,545	6.7	5.6	5.1	4.2
Grade 6 [12.08(0.59)]	5991	8.7	8.1	5.9	5.7
Grade 7 [13.02(0.60)]	7031	11.8	12.7	8.9	9.9
Grade 8 [13.95(0.62)]	4832	15.5	14.2	12.3	11.1
*χ*^2^		333.64^***^	406.88^***^	289.72^***^	348.35^***^
School types					
Public school	13,934	9.5	8.9	7.0	6.8
Private school	14,464	10.3	9.8	7.9	7.5
*χ*^*2*^		5.83^*^	6.87^**^	7.67^**^	5.84^*^
Boarding at school					
Yes	2317	11.3	10.4	7.9	7.8
No	26,081	9.8	9.3	7.4	7.1
*χ*^2^		5.34^*^	3.25	0.82	1.84
Ethnicity					
Han	27,055	9.9	9.4	7.4	7.1
Others	1343	9.8	9.2	7.4	6.6
*χ*^2^		0.01	0.03	0.01	0.68
Only-children family					
Yes	5638	8.8	8.5	7.2	6.3
No	22,760	10.2	9.6	7.5	7.3
*χ*^2^		9.45^**^	6.65^**^	0.70	7.47^**^
Parental marital status					
Good	27,047	9.6	9.1	7.2	6.9
Poor	1351	16.4	14.4	11.6	11.3
*χ*^2^		66.55^***^	41.88^***^	36.30^***^	38.00^***^
Family income (monthly)					
< ¥12,000	12,506	9.7	9.2	7.1	6.9
¥12,000–¥30,000	7423	8.9	8.9	6.8	7.0
> ¥30,000	2342	8.7	8.8	6.5	6.4
Unknown	6127	12.0	10.5	9.2	7.9
*χ*^2^		43.17^***^	13.11^**^	38.10^***^	8.06^*^
Father’s education					
Junior high school or less	8556	10.7	9.6	7.8	7.4
Senior high school	8459	9.2	9.4	7.2	6.7
College or more	11,383	9.8	9.2	7.3	7.2
*χ*^2^		11.45^**^	1.19	2.15	3.17
Mother’s education					
Junior high school or less	10,236	10.5	9.4	7.7	7.2
Senior high school	7905	9.3	9.7	7.4	7.3
College or more	10,257	9.7	9.0	7.2	6.9
*χ*^2^		6.80^*^	2.66	2.14	1.19
Chronic physical illness					
Yes	984	15.1	14.3	14.3	11.7
No	27,414	9.1	7.2	7.2	7.0
*χ*^2^		64.10^***^	40.24^***^	70.64^***^	32.21^***^
Family history of mental disorders					
Yes	267	24.0	20.2	21.0	12.4
No	28,131	9.8	9.3	7.3	7.1
*χ*^2^		59.94^***^	37.54	71.95	11.21^**^

All demographics variables were measured at Time 1

*p < 0.05, **p < 0.01, **p < 0.001

### The impact of the “Double Reduction” policy

**Fig. 1:**
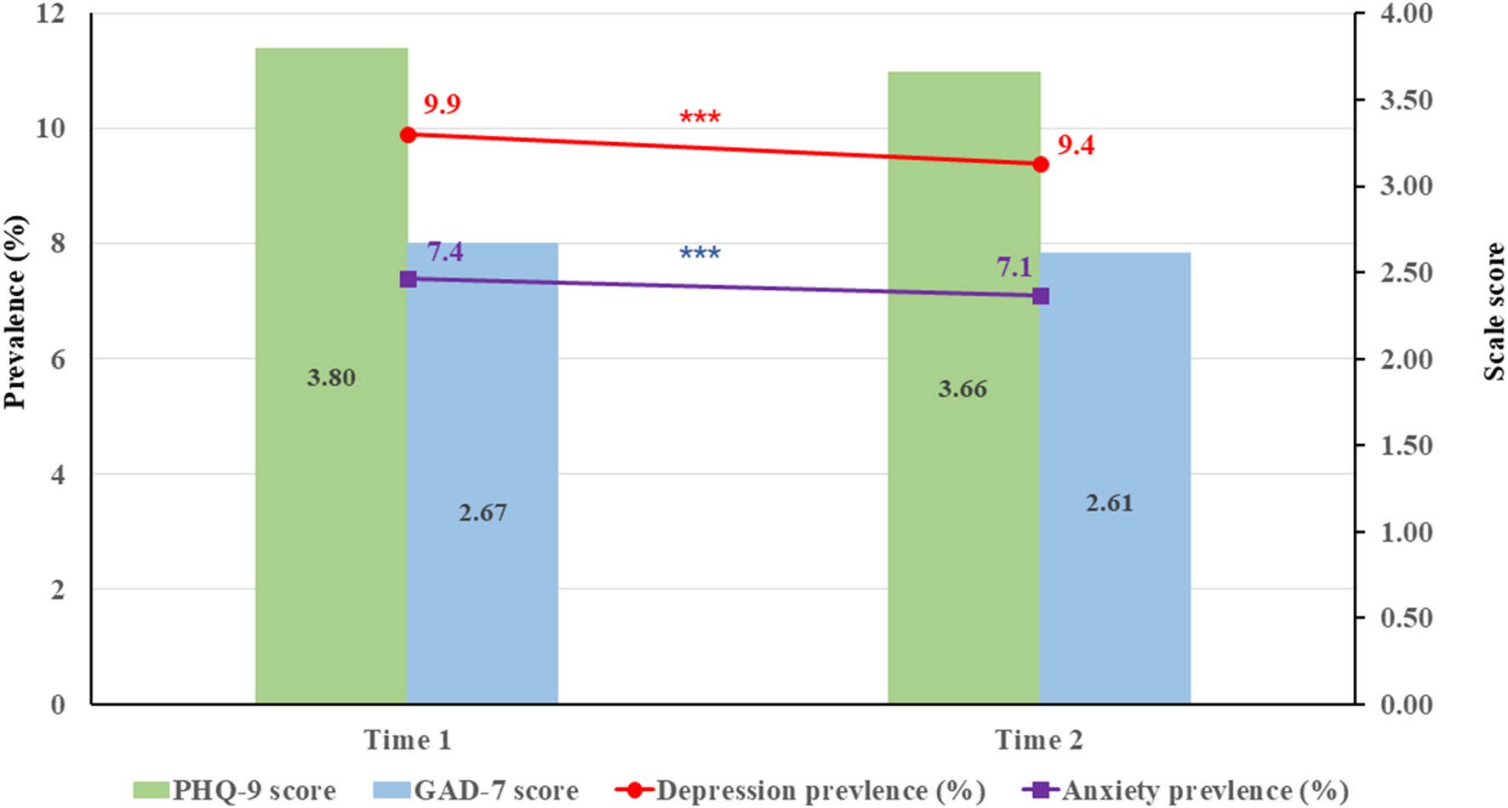
Prevalence rates and mean scores of depression and anxiety in two time points, ***p < 0.001

The PHQ-9 scores before the “Double Reduction” Policy were slightly higher than that of after the “Double Reduction” Policy (t = 4.92, p < 0.001, Cohen’s d = 0.030). Similar findings were observed in GAD-7 scores (t = 2.44, p = 0.015, Cohen’s d = 0.015). The prevalence of depressive and anxiety symptoms at T1 were 9.9% and 7.4% respectively, while rates slightly decreased at T2 (see Fig. 1). We further compared differences in the prevalence of depressive and anxiety symptoms at each wave in demographic characteristics (see Table 1).

### Changing patterns of depression and anxiety

**Fig. 2:**
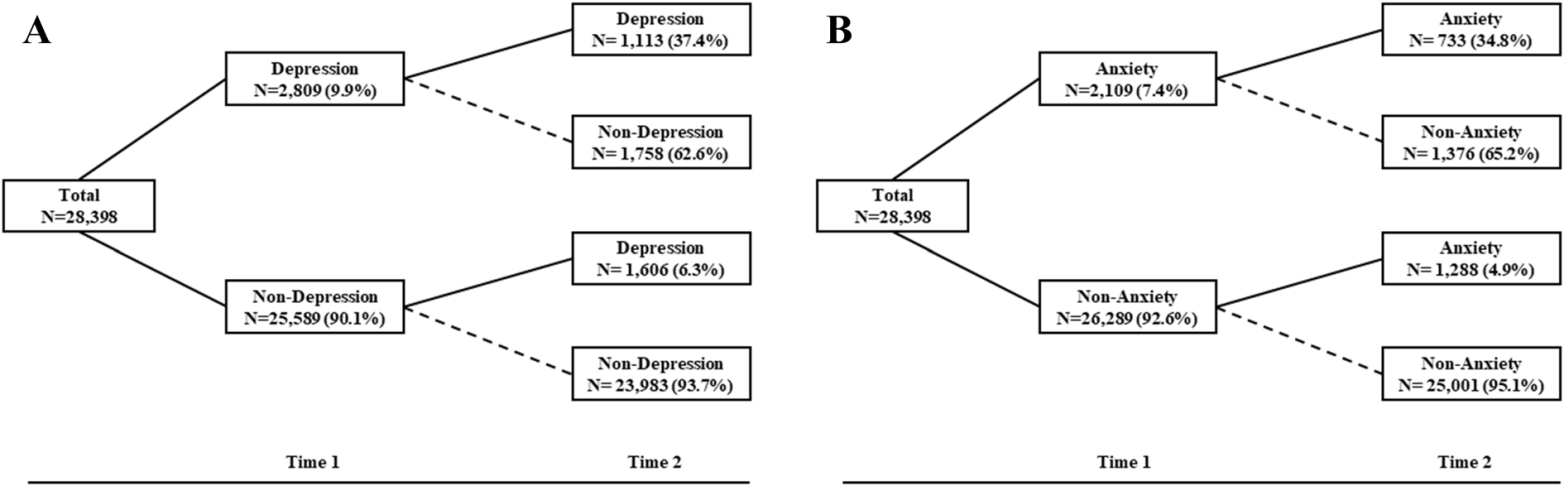
Change patterns of mental health problems. **A** Change patterns of depressive symptoms; **B** change patterns of anxiety symptoms

**Fig. 3:**
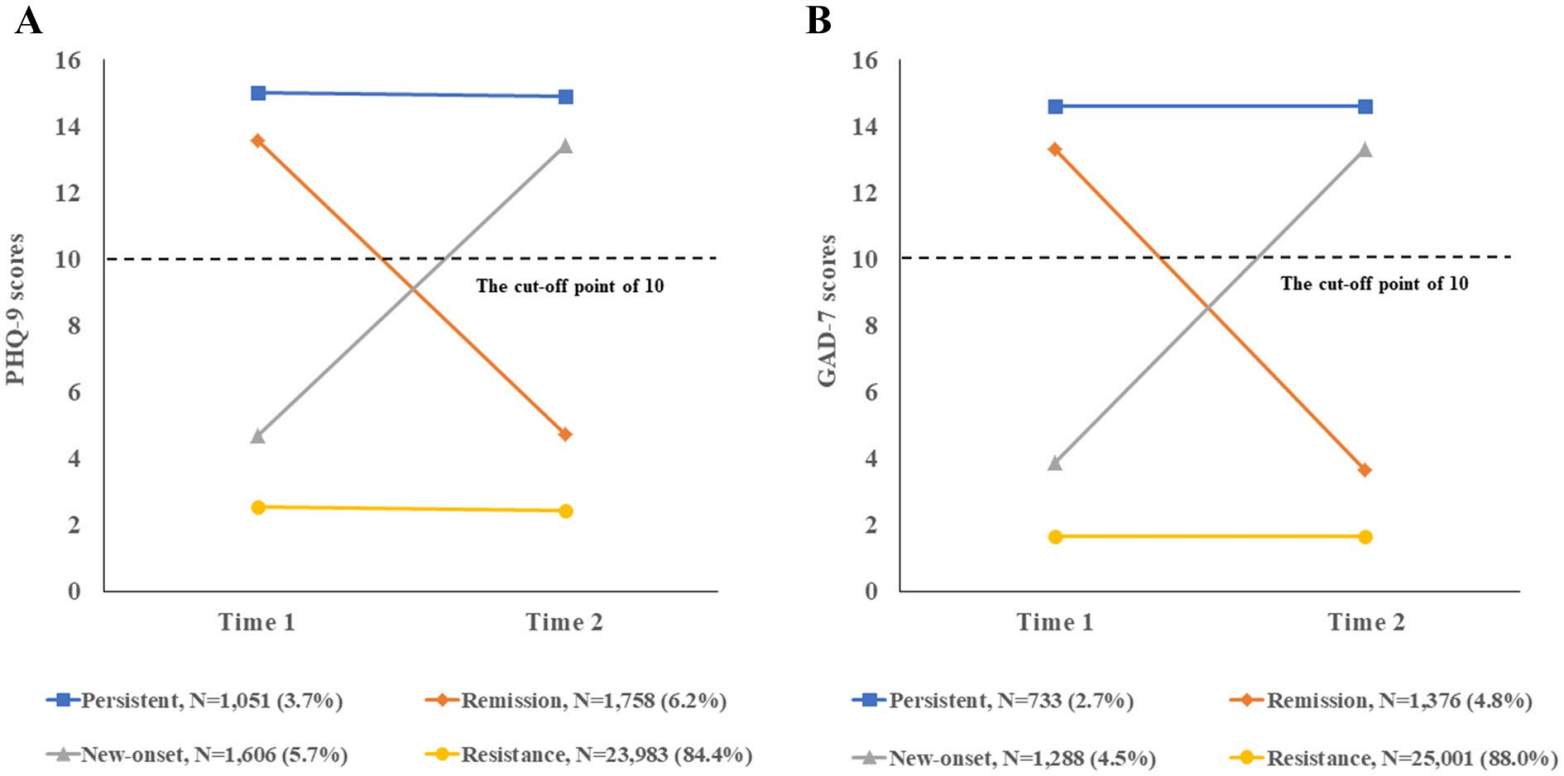
Trajectories of mental health problems. **A** Trajectory for depressive symptoms. **B** Trajectory for anxiety symptoms

The changing patterns of depressive symptoms were presented in Fig. 2A. We further identified four groups of symptoms trajectory in Fig. 3A. The persistent group (3.7%, N = 1051): met criteria (PHQ-9 score ≥ 10) at both waves; the resistance group: 84.4% (N = 23,983) of participants did not meet criteria for depressive symptoms (i.e., PHQ-9 score < 10). The new-onset group: 5.7% of the sample (N = 1606) did not report depressive symptoms at T1 but had at T2. The remission group (N = 1758, 6.2%): participants reported depressive symptoms at T1 but recovered at T2. As for the changing patterns of anxiety symptoms, we adopted the same grouping method (see Fig. 2B). As shown in Fig. 3B: persistent (N = 733, 2.7%), remission (N = 1376, 4.8%), new-onset (N = 1288, 4.5%), resistance (N = 25,001, 88.0%).

### Predictors of changing patterns of depression/anxiety

As shown in Table 2, girls were more likely to show new-onset and persistent depressive or anxiety symptoms. The likelihood of developing new-onset depressive symptoms increased if the participants have a family history of mental disorders. Grade 6 students had lower odds of new-onset anxiety symptoms than other graders. Meanwhile, several predictors may also suggest a potential effect on depressive and anxiety symptoms but *p* values at a 0.01 level. For example, students reporting poor parental marital status were more likely to develop persistent depressive and anxiety symptoms. Those had a chronic physical illness may be at higher risk of experiencing new-onset depressive and anxiety symptoms.

**Table 2:** Risk and protective factors of depression or anxiety new-onset [OR(95%CI)]

	Depression		Anxiety	
	New-onset vs Resistance	Persistent vs Remission	New-onset vs Resistance	Persistent vs Remission
Measured at T1				
Girls (boys as Ref.)	1.40 (1.26, 1.56)^***^	1.59 (1.34, 1.88)^***^	1.49 (1.32, 1.68)^***^	1.64 (1.34, 1.99)^***^
Grade (grade 8 as Ref.)				
Grade 5	0.80 (0.68, 0.94)^**^	0.84 (0.67, 1.08)	0.85 (0.71, 1.03)	0.80 (0.60, 1.05)
Grade 6	0.74 (0.62, 0.87)^**^	0.72 (0.56, 0.91)^**^	0.70 (0.58, 0.84)^***^	0.63 (0.47, 0.85)^**^
Grade 7	1.01 (0.87, 1.17)	1.02 (083, 1.26)	1.09 (0.92, 1.28)	0.91 (0.72, 1.16)
Private school (Public school as Ref.)	1.10 (0.98, 1.25)	1.21 (1.01, 1.46)^*^	1.16 (1.01, 1.33)^*^	1.05 (0.85, 1.30)
Boarding at school (No as Ref.)	1.04 (0.86, 1.27)	0.65 (0.47, 0.88)^**^	0.99 (0.79, 1.23)	0.68 (0.47, 0.98)^*^
Ethnicity Han (Others as Ref.)	0.98 (0.76, 1.26)	0.83 (0.57, 1.19)	0.93 (0.70, 1.23)	1.24 (0.79, 1.96)
Only-children family (No as Ref.)	0.93 (0.81, 1.07)	0.97 (0.78, 1.20)	0.86 (0.73, 1.01)	0.94 (0.73, 1.19)
Poor parental marital status (Good as Ref.)	1.11 (0.88, 1.41)	1.51 (1.12, 2.02)^**^	1.24 (0.97, 1.60)	1.64 (1.17, 2.32)^**^
Family income (monthly) (< ¥12,000 as Ref.)				
¥12,000–¥30,000	0.91 (0.79, 1.05)	1.11 (0.89, 1.37)	0.93 (0.80, 1.09)	1.15 (0.90, 1.48)
> ¥30,000	0.92 (0.74, 1.15)	1.20 (0.86, 1.66)	0.95 (0.75, 1.21)	0.84 (0.57, 1.26)
Unknown	–	–	–	–
Father’s education (College or more as Ref.)				
Junior high school or less	1.06 (0.89, 1.25)	0.94 (0.73, 1.20)	1.01 (0.84, 1.21)	0.89 (0.67, 1.19)
Senior high school	1.04 (0.90, 1.20)	1.06 (0.85, 1.32)	0.90 (0.77, 1.06)	0.89 (0.69, 1.15)
Mother’s education (College or more as Ref.)				
Junior high school or less	0.85 (0.80, 1.11)	1.12 (0.88, 1.42)	1.00 (0.83, 1.20)	1.02 (0.77, 1.36)
Senior high school	1.04 (0.90, 1.20)	1.18 (0.95, 1.47)	1.03 (0.88, 1.22)	1.17 (0.90, 1.50)
Chronic physical illness (No as Ref.)	1.49 (1.16, 1.91)^**^	1.11 (0.80, 1.54)	1.46 (1.11, 1.92)^**^	1.06 (0.73, 1.53)
Family history of mental disorders (No as Ref.)	2.30 (1.50, 3.53)^***^	1.09 (0.64, 1.84)	1.31 (0.75, 2.28)	0.79 (0.44, 1.45)
Measured at T2				
Negative life events (contact variable)	1.03 (1.03, 1.04)^***^	1.03 (1.03, 1.03)^***^	1.04 (1.04, 1.04)^***^	1.03 (1.03, 1.04)^***^
Sleep duration > 8 h/n (No as Ref.)	0.33 (0.26, 0.43)^***^	0.38 (0.25, 0.57)^***^	0.35 (0.27, 0.47)^***^	0.46 (0.29, 0.75)^***^
Reduced homework (No as Ref.)	0.78 (0.68, 0.89)^***^	0.85 (0.70, 1.04)	0.77 (0.67, 0.89)^***^	0.81 (0.65, 1.02)
More extracurricular activities (No as Ref.)	0.77 (0.66, 0.88)^***^	0.84 (0.68, 1.04)	0.73 (0.63, 0.86)^***^	0.71 (0.55, 0.91)^**^
Increased physical activity (No as Ref.)	0.98 (0.85, 1.11)	0.90 (0.74, 1.10)	1.01 (0.87, 1.16)	1.08 (0.86, 1.36)
More time with parents (No as Ref.)	0.52 (0.46, 0.59)^***^	0.58 (0.48, 0.71)^***^	0.48 (0.41, 0.55)^***^	0.71 (0.57, 0.90)^**^
Reduced academic stress (No as Ref.)	0.59 (0.51, 0.68)^***^	0.60 (0.48, 0.75)^***^	0.50 (0.42, 0.59)^***^	0.45 (0.34, 0.59)^***^

Bold: *p* < 0.001 and OR > 1.2 (or < 0.9) were considered to have scientific and public health significance

Furthermore, changes in lifestyles after the implementation of “Double Reduction” Policy have significant effects on develop depressive and anxiety symptoms after controlling for demographics and negative life events. Specifically, reduced homework, more extracurricular activities, more time with parents, and reduced academic pressure were protective factors against poor mental health. In addition, students with sleep duration > 8 h per night were less likely to have new-onset and persistent mental symptoms in comparison to those who reported sleep duration ≤ 7 h per night.

## Discussion

This is the first study to explore the impact of the “Double Reduction” Policy on mental health among adolescents in China. We examined changing patterns of depressive and anxiety symptoms before and after the “Double Reduction” Policy. Four different patterns were identified. We further explore the influential factors of these patterns.

It is noteworthy that the prevalence of depression (9.9% vs. 9.4%) and anxiety (7.4% vs. 7.1%) slightly decreased after the policy was implemented. These findings indicated that the “Double Reduction” Policy might relieve adolescents’ mental health, echoing the literature that academic burden negatively impacts on mental health [9, 25]. We acknowledge that this decreasing trend is small. Several assumptions might help us understand this atmosphere. First, the implementation of the “Double Reduction” Policy is still in its early stage. It is possible that the obvious positive impacts will be observed with long-term multiple follow-ups. Second, lifestyle changes caused by COVID-19 pandemic (e.g., restrictions on social activities, online classes) may adversely affect adolescents’ mental health [26, 27]. However, it is a pity that we did not have available data to tease out its impacts. These reasons may influence the effectiveness of the “Double Reduction” Policy.

The changing patterns of depressive and anxiety symptoms showed that the majority of students exhibited very mild or no mental health at two surveys (84.4% and 88.0% for resistance of depressive and anxiety symptoms respectively). Previous studies also suggested that approximately more than half of the children and adolescents maintain a stable healthy functioning over time [28]. Costello and colleagues found that about 28.7% of adolescents reported non-depressed mood symptoms and 59.4% had low symptoms patterns across 6 years [29]. Moreover, a very small percentage of children and adolescents have persistent (all rates were about 3%) or new-onset (all rates were about 5%) mental health problems in the current study. The potential explanation for those who suffer from persistent or new-onset mental health problems, as follows: Without additional support from off-campus training courses, some students may be concerned about their grades. Meanwhile, an increase in extracurricular activities increases the risk of some students’ excessive Internet use [30], which may also affect their mental health.

We found that participants who had reduced academic stress after the “Double Reduction” Policy were less likely to report persistent and new-onset mental health problems than those who had not. Greenberger and colleagues have found that the association between academic stress and poor mental health appears stronger among Chinese students than those in western countries [31]. Academic stress is associated with the notably high learning/testing standards, outcomes, and expectations of secondary schools in China, which may play a significant role in explaining high incidences of anxiety and depression among Chinese adolescents [32]. Our analyses of the “Double Reduction” Policy related measures found that reduced homework and more extracurricular activities were protective against depressive and anxiety symptoms. Previous work has indicated that adolescents who spent long hours on homework reported higher depression level [9]. Longer homework/studying durations were associated with less nocturnal sleep and greater academic stress [6, 7], leading to increased anxious symptoms [9]. In addition, we found participation in extracurricular activities could improve adolescents’ health, which is consistent with the literature [33, 34].

Results in the logistic model showed that those who had sleep duration more than 8 h per night may be less likely to report new onset and persistent mental health symptoms. Consistent with the literature [35, 36], insufficient sleep can disturb an individual’s emotional regulation, which may increase depressive and anxiety symptoms. Furthermore, coincides with previous findings [37], adolescents who spend more time with their parents are less likely to have persistent and new onset mental health problems prospectively. Parental presence may provide continuous support to the adolescent when she/he encounters challenges. It is also possible that engaging in tasks together with parents may help to minimize adolescents’ ruminative processes and facilitate greater behavioral activation; these two factors have been identified as potential mechanisms of psychotherapy for depression [38, 39]. When considering the influence of demographic factors, our results echo previous findings that girls [40], poor parental marital status [41], chronic physical illness [42], and family history of mental disorders [43] were associated with poor mental health. Students in Grade 6 have a lower risk of new-onset anxiety, which may be explained by the fact that they were in the first stage of junior high school when we conducted our second survey. At that time, they had already passed the junior high school entrance examination and thus their study pressure was relatively lower. We suggest future psychosocial interventions in adolescents may need to take these factors into consideration.

Several limitations need to be acknowledged. Firstly, depressive and anxiety symptoms were assessed by self-report questionnaires rather than clinical interviews, as well as two surveys were administered using a web-based questionnaire, which may result in potential reporting bias. Second, there was a high attrition rate, which may impact the accuracy of changes in symptoms. In the second survey (December 2021), students in grades 6 entered junior high schools through the entrance examination, and only a small number of students who stayed in the local school participated in the second survey. Although mild significant differences with small effect sizes (Cramer’s V = 0.012 and 0.022) were found for the baseline prevalence between participants who were followed up and those lost to follow-up, the results need to be interpreted with caution. Third, we used only dichotomous variables to measure lifestyle change, which provides a limited interpretation of the specific impact of the “Double Reduction” Policy on students’ learning and lives. Furthermore, the duration between two waves was 8 months, which only reflect a temporary change before and after the “Double Reduction” Policy. To our knowledge, there are no other studies to examine the effect of the “Double Reduction” Policy on students’ mental health, and our study is the first evaluation of the effect of the policy on students’ well-being. Longer follow-ups are needed to explore the long-term effects of this policy implementation on students’ well-being.

## Conclusions

A declined trend in depression and anxiety among Chinese adolescents was observed after the “Double Reduction” Policy. More attention needs to be drawn to those at higher risk of developing persistent or new-onset mental health problems. In addition, developing psychological interventions aiming at increasing sleep duration, extending time with parents, and taking part in extracurricular activities, as well as reducing homework load and academic pressure is critical for the prevention of essential mental health problems.

## Data Availability

The dataset used and/or analyzed during the current study are available from the corresponding author (FF) on reasonable request.
